# Supplementary material for: Diffusion MRI processing in the HEALthy Brain and Child Development study: Innovations and applications
Source: Dev Cogn Neurosci. 2026 Oct 15;82:101838. doi: 10.1016/j.dcn.2026.101838 (PMC13734816; doi:10.1016/j.dcn.2026.101838)
Supplement: Supplementary file 1 [file mmc10.pdf]

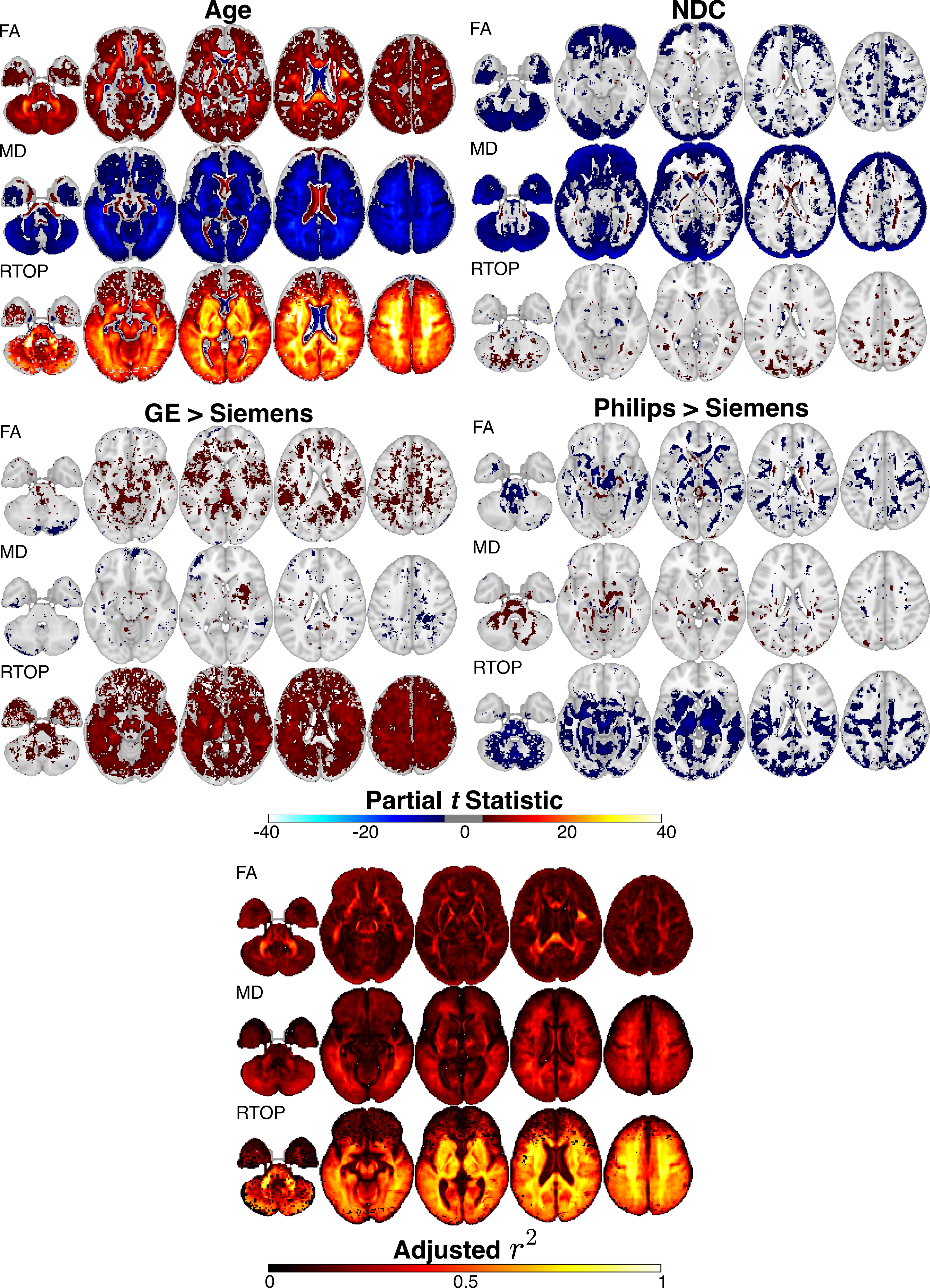

Age
NDC
GE > Siemens
Philips > Siemens
FA
MD
RTOP
Partial t Statistic
-40
-20
0
20
40
Adjusted r^2
0
0.5
1
