# Supplementary material for: Diffusion MRI processing in the HEALthy Brain and Child Development study: Innovations and applications
Source: Dev Cogn Neurosci. 2026 Oct 15;82:101838. doi: 10.1016/j.dcn.2026.101838 (PMC13734816; doi:10.1016/j.dcn.2026.101838)
Supplement: Supplementary file 2 [file mmc11.pdf]

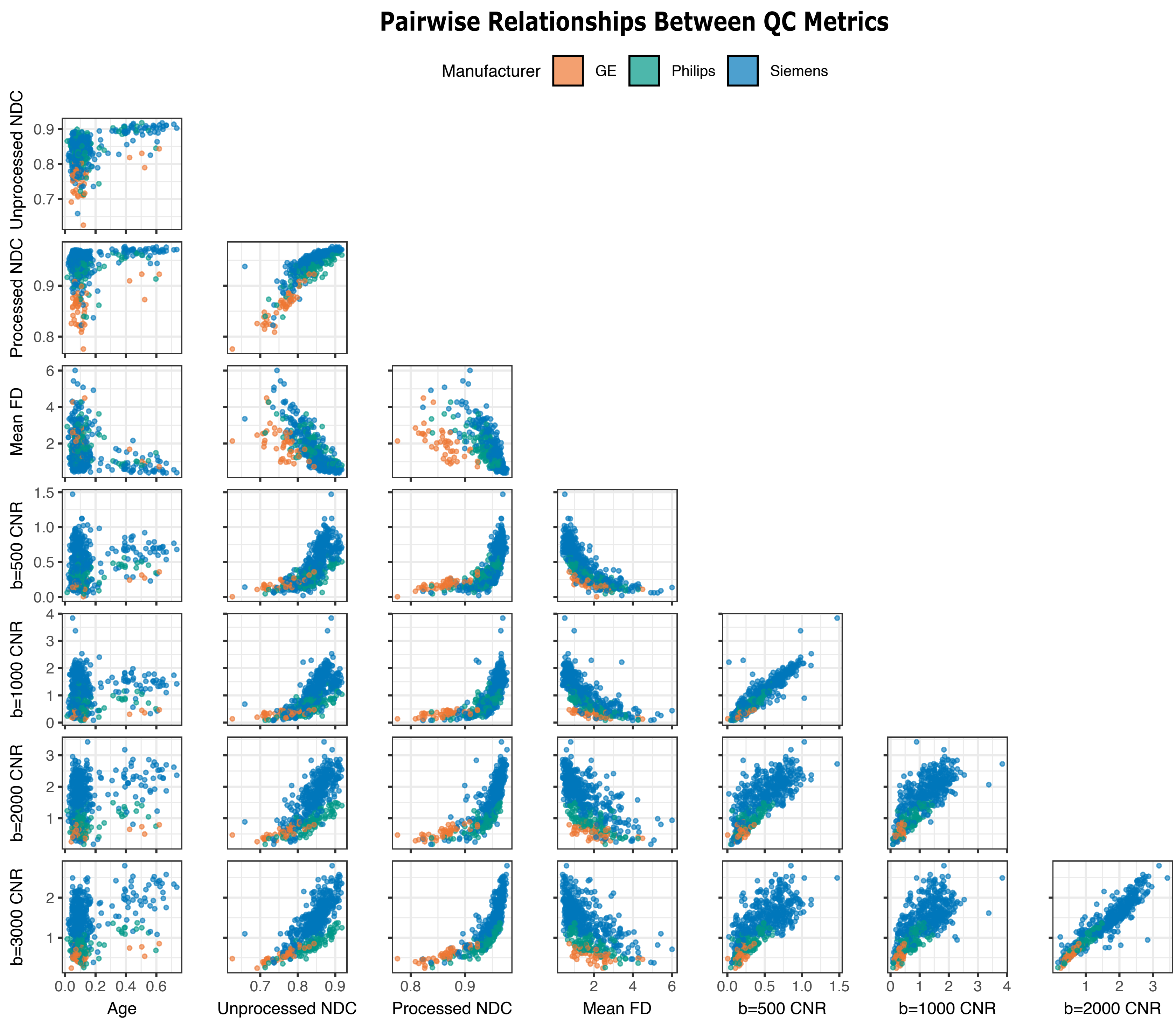

Pairwise Relationships Between QC Metrics
Manufacturer
GE
Philips
Siemens
Unprocessed NDC
Processed NDC
Mean FD
b=500 CNR
b=1000 CNR
b=2000 CNR
b=3000 CNR
Age
