# Supplementary material for: Diffusion MRI processing in the HEALthy Brain and Child Development study: Innovations and applications
Source: Dev Cogn Neurosci. 2026 Oct 15;82:101838. doi: 10.1016/j.dcn.2026.101838 (PMC13734816; doi:10.1016/j.dcn.2026.101838)
Supplement: Supplementary file 3 [file mmc12.pdf]

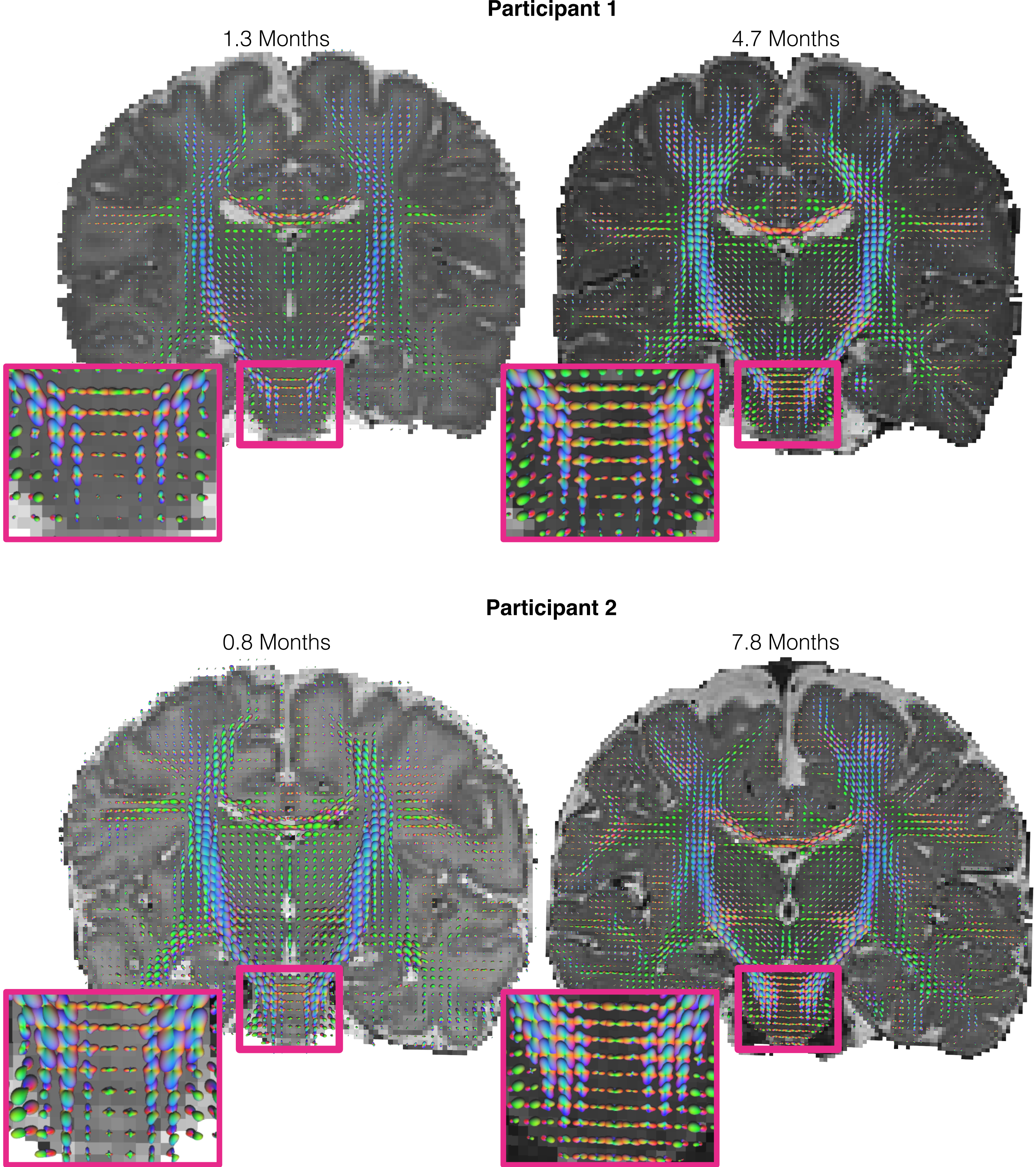
Participant 1
1.3 Months
4.7 Months
Participant 2
0.8 Months
7.8 Months
