# Supplementary material for: Diffusion MRI processing in the HEALthy Brain and Child Development study: Innovations and applications
Source: Dev Cogn Neurosci. 2026 Oct 15;82:101838. doi: 10.1016/j.dcn.2026.101838 (PMC13734816; doi:10.1016/j.dcn.2026.101838)
Supplement: Supplementary file 4 [file mmc5.pdf]

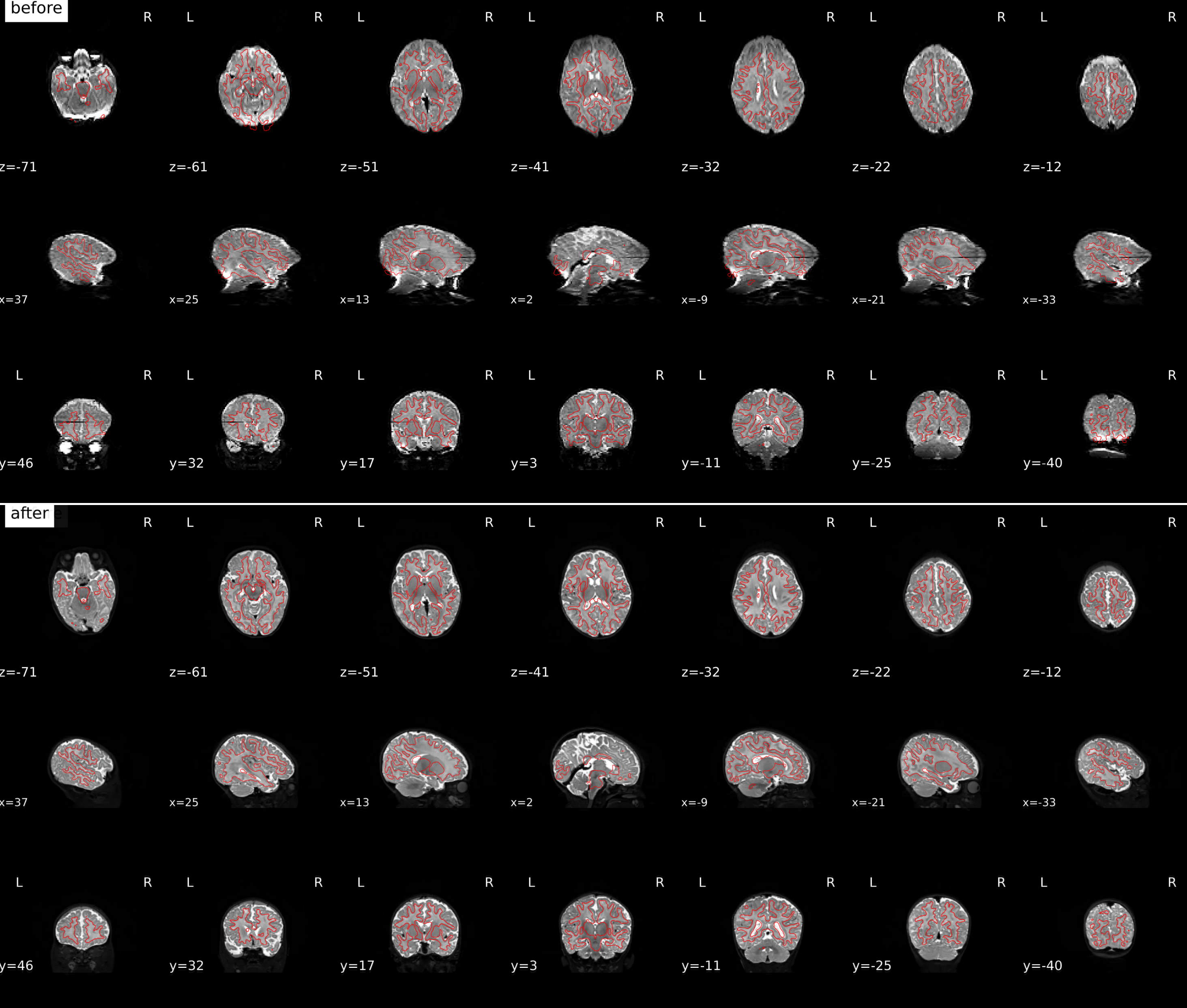

before
L R L R L R L R L R L R L R
z=-71 z=-61 z=-51 z=-41 z=-32 z=-22 z=-12
x=37 x=25 x=13 x=2 x=-9 x=-21 x=-33
L R L R L R L R L R L R L R
y=46 y=32 y=17 y=3 y=-11 y=-25 y=-40
after
L R L R L R L R L R L R L R
z=-71 z=-61 z=-51 z=-41 z=-32 z=-22 z=-12
x=37 x=25 x=13 x=2 x=-9 x=-21 x=-33
L R L R L R L R L R L R L R
y=46 y=32 y=17 y=3 y=-11 y=-25 y=-40
