# Supplementary material for: Diffusion MRI processing in the HEALthy Brain and Child Development study: Innovations and applications
Source: Dev Cogn Neurosci. 2026 Oct 15;82:101838. doi: 10.1016/j.dcn.2026.101838 (PMC13734816; doi:10.1016/j.dcn.2026.101838)
Supplement: Supplementary file 5 [file mmc6.pdf]

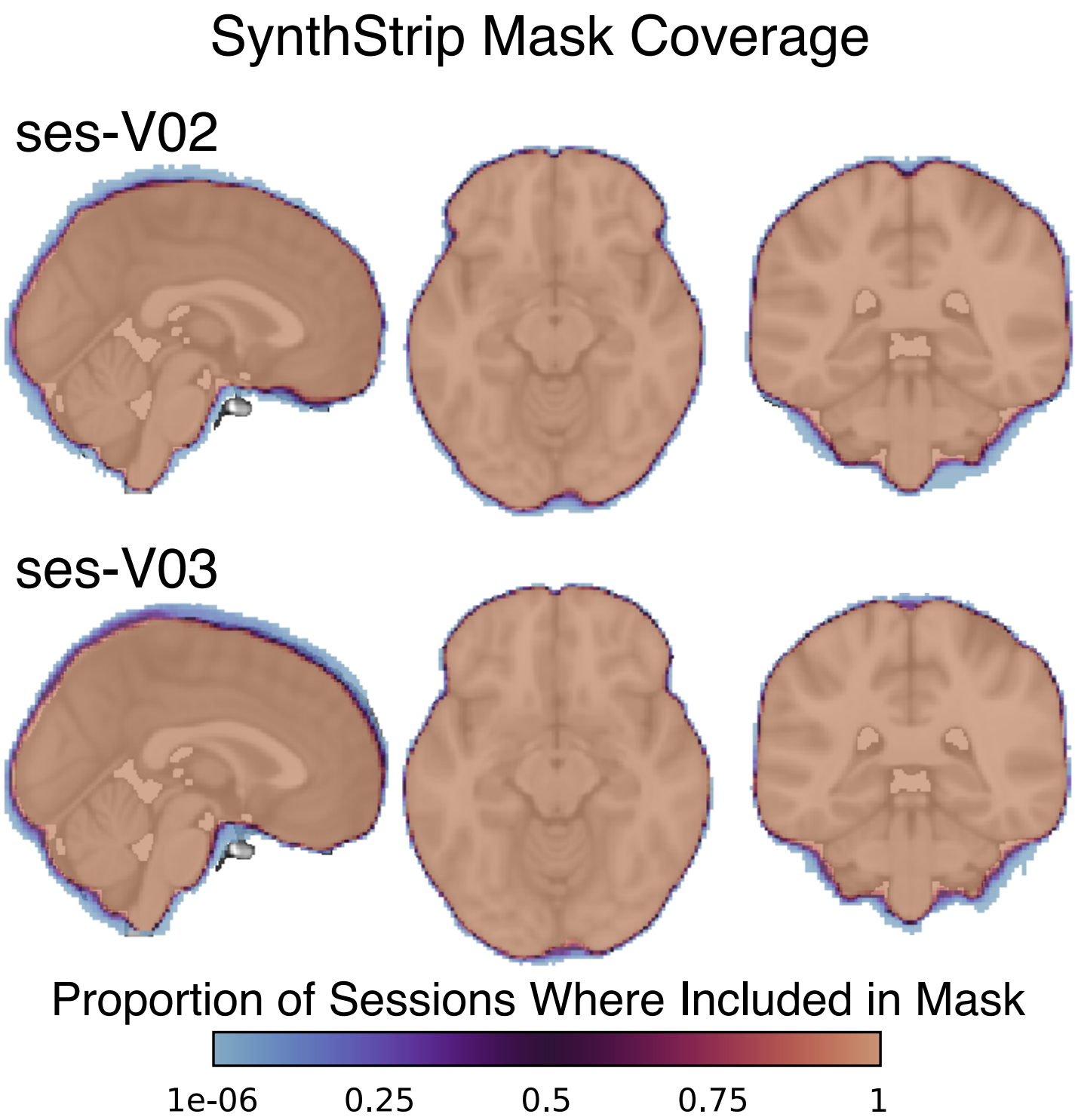
SynthStrip Mask Coverage
ses-V02
ses-V03
Proportion of Sessions Where Included in Mask
1e-06
0.25
0.5
0.75
1
