# Supplementary material for: Diffusion MRI processing in the HEALthy Brain and Child Development study: Innovations and applications
Source: Dev Cogn Neurosci. 2026 Oct 15;82:101838. doi: 10.1016/j.dcn.2026.101838 (PMC13734816; doi:10.1016/j.dcn.2026.101838)
Supplement: Supplementary file 6 [file mmc7.pdf]

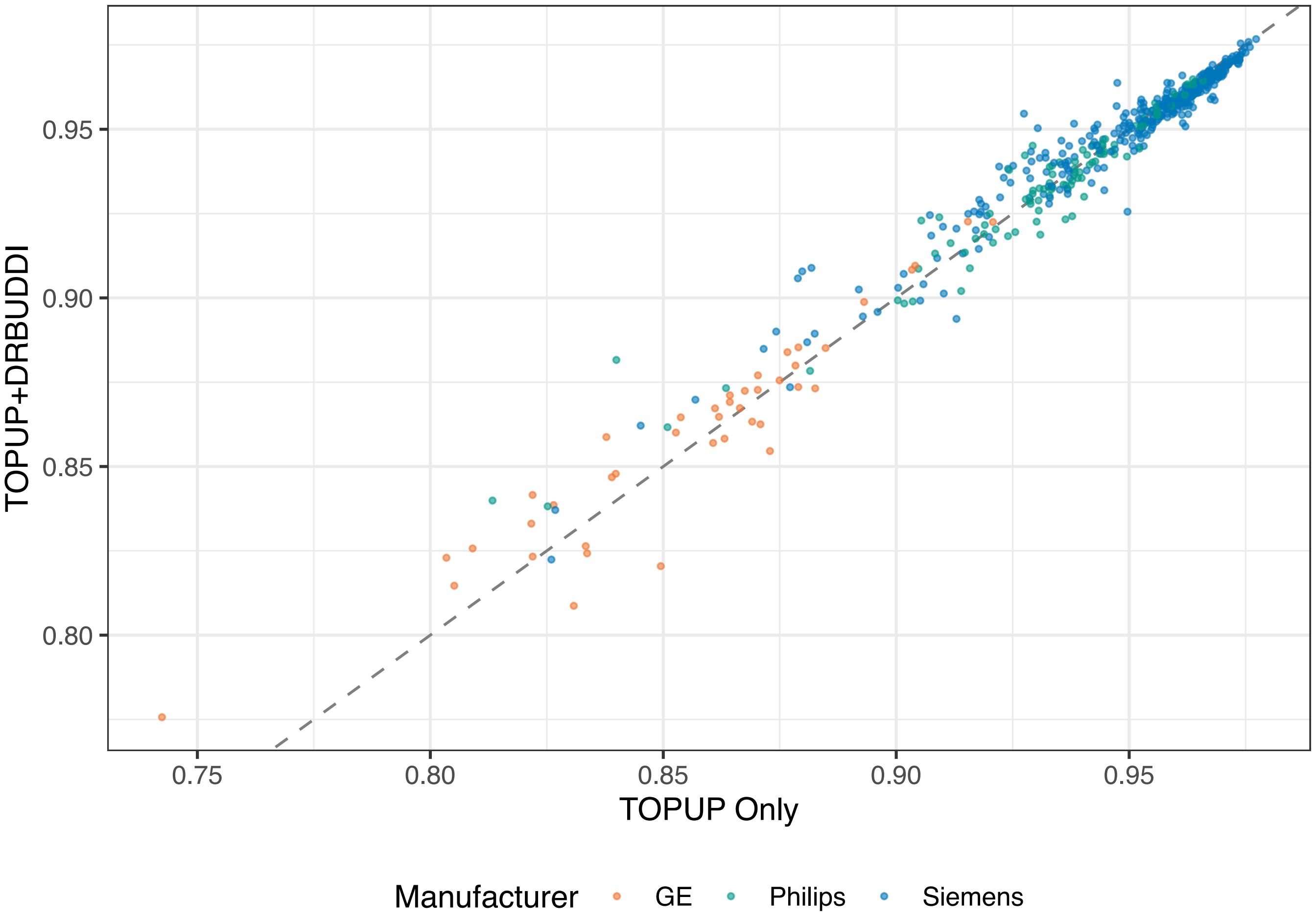

TOPUP+DRBUDDI
0.95
0.90
0.85
0.80
0.75
0.80
0.85
0.90
0.95
TOPUP Only
Manufacturer
GE
Philips
Siemens
