# Supplementary material for: Diffusion MRI processing in the HEALthy Brain and Child Development study: Innovations and applications
Source: Dev Cogn Neurosci. 2026 Oct 15;82:101838. doi: 10.1016/j.dcn.2026.101838 (PMC13734816; doi:10.1016/j.dcn.2026.101838)
Supplement: Supplementary file 8 [file mmc9.pdf]

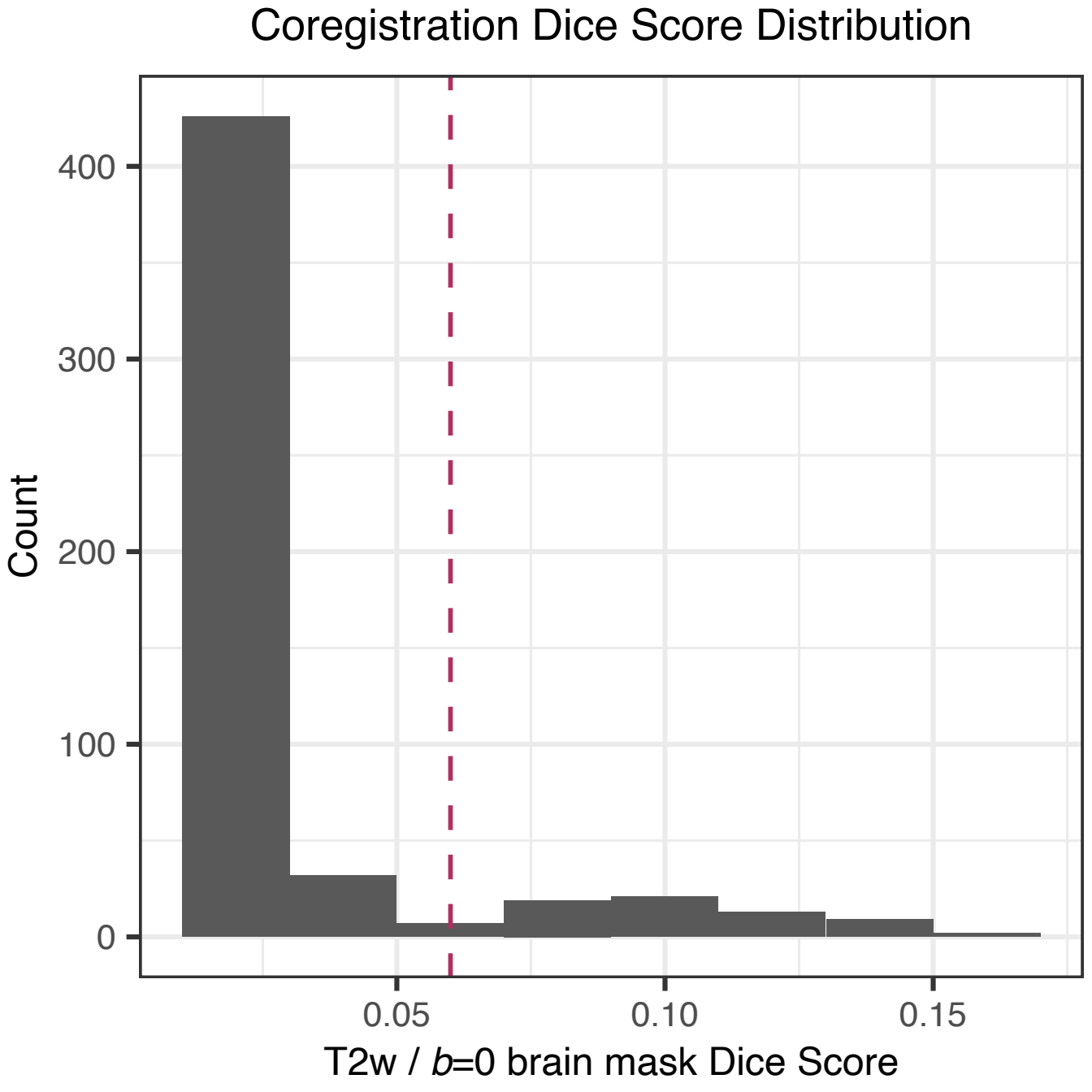

Coregistration Dice Score Distribution
Count
400
300
200
100
0
0.05
0.10
0.15
T2w / b=0 brain mask Dice Score
