# Supplementary material for: Diffusion MRI processing in the HEALthy Brain and Child Development study: Innovations and applications
Source: Dev Cogn Neurosci. 2026 Oct 15;82:101838. doi: 10.1016/j.dcn.2026.101838 (PMC13734816; doi:10.1016/j.dcn.2026.101838)
Supplement: Supplementary file 9 [file mmc1.docx]

# SUPPLEMENTARY MATERIAL

**Supplementary Table 1***:* Sources of the sessions from the HBCD beta release. If a site upgraded their scanner OS or model, it appears in multiple rows (e.g., site35).

**Supplementary Table 2.** Parametric microstructure maps produced by *QSIRecon* from the hbcd_scalar_maps workflow. Where applicable, the categorization from Sadikov et al., (2025) is included as a guideline for general interpretation of each parameter (each Category is shaded with a different color).

**Supplementary Table 3.** Model-specific CNR values for each shell.

**Supplementary Table 4.** The 67 white matter bundles returned by the hbcd_scalar_maps workflow, grouped by the AutoTrack category used to request them (Association, Commissure, Cerebellum, and the basal ganglia and brainstem subdivisions of Projection). Bundles marked (L, R) are estimated separately in each hemisphere; the remainder are midline or interhemispheric and are estimated once. Figures 9 and 10 collapse the bilateral bundles across hemispheres. In the released tabular data each bundle is named by its category and label without spaces, with a trailing L or R where applicable (e.g., Association_ArcuateFasciculusL, Cerebellum_Vermis). The dentatorubrothalamic tract is labeled by the hemispheres in which it begins and ends (-lr, -rl) rather than by a single hemisphere, as in Figure 9.

## Figures

**Supplementary Figure 1.** An example visual report of the *SynthSeg* white matter segmentation overlaid on a *b*=0 image before and after correction by *TOPUP*. While not used quantitatively in *QSIPrep* or *QSIRecon*, the segmentation is a useful guide to visually inspect the accuracy of SDC.

**Supplementary Figure 2**. The coverage map of infant T2w-based *SynthStrip* masks mapped to MNINLin6Asym space. The highest variability of whether a voxel is masked or not is in a tight ribbon around the cortical surface. Both sessions V02 and V03 show high agreement on which voxels are within the brain.

**Supplementary Figure 3.** There is no significant difference in NDC when including DRBUDDI in preprocessing paired *t*(528) = -0.94 *n.s*.

**Supplementary Figure 4.** The adjusted $r^{2}$ for the model fits displayed in **Figure 7** are shown for FA, MD and RTOP. The adjusted r2 is calculated by R’s lm() function, which penalizes the inclusion of model parameters. Note that the design matrix fit in each of these models is identical, including NDC, scanning site, vendor and corrected age at scan to predict FA, MD or RTOP.

**Supplementary Figure 5.** Following coregistration and resampling, a Dice distance was calculated based on the SynthSeg brain masks based on the T2w and mean *b*=0 images from each session. Higher Dice distances indicate lower overlap between the masks and poor coregistration. A vertical dashed line at $x=0.06$ reflects the cutoff selected for inclusion in the mass univariate analysis in **Figure 7**.

**Supplementary Figure 6.** Statistical tests including all data (*n*=529). Including sessions with high coregistration Dice distance reduces overall $r^{2}$ and specifically impacts the RTOP model fits in the front of the brain.

**Supplementary Figure 7.** The relationship between age, head motion and automated QC metrics from *QSIPrep* shows that mean framewise displacement (FD) is highly correlated with both unprocessed and processed NDC. The NDC measures are also highly correlated with CNR, which also correlates with itself at each of the different shells.

**Supplementary Figure 8.** Diffusion ODFs and major white matter bundles plotted for two longitudinal participants. Each row shows an expanded view of the pons (inset) and a coronal slice of the T2w image with dODFs plotted as scaled 3D glyphs. Size and brightness of the glyphs are scaled by anisotropy. The ses-V02 dODFs are less anisotropic as expected, with ses-V03 dODF glyphs both larger and brighter. Notably, crossings are visible in the dODFs in the pons in both sessions for both participants in the inset.

## Supplementary Methods (Automated Boilerplate)

Preprocessing was performed using *QSIPrep* 1.0.0 (Cieslak et al., 2021), which is based on *Nipype* 1.9.1 [(Gorgolewski et al., 2011); RRID:SCR_002502]. Many internal operations of *QSIPrep* use *Nilearn* 0.10.1 (Abraham et al., 2014) and *Dipy* (Garyfallidis et al., 2014).

The T2-weighted anatomical reference image was reoriented into AC-PC alignment via a 6-DOF transform extracted from a full affine registration to the MNIInfant template appropriate for the participant’s age. A full nonlinear registration to the template from AC-PC space was estimated via symmetric nonlinear registration (SyN) using antsRegistration. Brain extraction was performed on the T2w image using *SynthStrip* (Hoopes et al., 2022) and automated segmentation was performed using *SynthSeg* (Billot et al., 2023) from *FreeSurfer* version 7.3.1.

Initial denoising steps were applied to the AP and PA acquisitions *separately.* DWI data were denoised using the Marchenko-Pastur PCA method implemented in *dwidenoise* (Tournier et al. 2019; Veraart et al. 2016; Veraart, Fieremans, and Novikov 2016; Cordero-Grande et al. 2019) with a window size of 3 voxels. After MP-PCA, Gibbs ringing was removed using *TORTOISE* (H.-H. Lee et al., 2021). Any images with a b-value less than 100 s/mm^2^ were treated as a *b*=0 image. The mean intensity of the DWI series was adjusted so all the mean intensity of the *b*=0 images matched across the AP and PA scans. B1- (receive-field) inhomogeneity correction was not included during preprocessing because this artifact was corrected for on the scanner (i.e., Prescan Normalize, PURE, and CLEAR for Siemens, GE, and Philips, respectively). The AP and PA scans were then concatenated for processing with *eddy*.

*FSL* (version 6.0.7.8)’s *eddy* was used for head motion correction and *eddy* current correction (Andersson et al., 2016). *eddy* was configured with a *q*-space smoothing factor (a.k.a the “fudge factor”) of 10, a total of 5 iterations, and 1000 voxels used to estimate hyperparameters. The FWHM of the first iteration in *eddy* was increased to allow for larger head motion and potential large differences in head positioning between the AP and PA scans. A quadratic first level model was used to characterize *eddy* current-related spatial distortion. *q*-space coordinates were forcefully assigned to shells. Field offset was attempted to be separated from subject movement. Shells were aligned post-*eddy*. *eddy*’s outlier replacement was run (Andersson et al., 2016). Data were grouped by slice, only including values from slices determined to contain at least 250 intracerebral voxels. Groups deviating by more than 4 standard deviations from the prediction had their data replaced with imputed values.

Data was collected in a pair of scans with reversed phase-encoding directions, resulting in scans with distortions going in opposite directions. Distortion correction was performed in two stages. In the first stage, *FSL*’s *TOPUP* (Andersson et al., 2003) was used to estimate a susceptibility-induced off-resonance field based on *b*=0 images extracted from both DWI series (with reversed phase encoding directions). The *TOPUP*-estimated fieldmap was incorporated into the eddy current and head motion correction interpolation. Dynamic susceptibility distortion correction was applied with 10 iterations, lambda=10.00 and spline knot-spacing of 10.00 mm (Andersson et al., 2018). Interpolation after head motion and initial susceptibility distortion correction was performed using the jac method.

*DRBUDDI* (Irfanoglu et al., 2015), part of the *TORTOISE* (Irfanoglu et al., 2025) software package, was used to perform a second stage of distortion correction. *DRBUDDI* was run on data output by *eddy*: motion-corrected DWI series split by their phase encoding directions. Specifically, tensor-based synthetic *b*=0 and fractional anisotropy images are computed separately for each phase encoding direction. These are then used together with the T2-weighted structural image in a multimodal registration to estimate the residual susceptibility-induced off-resonance field not detected by TOPUP. Signal intensity was adjusted in the final interpolated images using a method similar to *eddy*’s Least Squares Reconstruction (LSR).

Several confounding time-series were calculated based on the preprocessed DWI: framewise displacement (FD) using the implementation in *Nipype* (following the definitions by (Power et al., 2014)) was calculated based on the motion parameters estimated by *eddy*. The head-motion estimates calculated in the correction step were also placed within the corresponding confounds file. Note that estimation of translation in the phase encoding axis has high uncertainty since that particular aspect of head motion is difficult to distinguish from the effect of a constant (mean) eddy-current field. Slicewise cross correlation was also calculated. QC metrics included in Richie-Halford et al. (2022) such as NDC and coherence index, were calculated along with some new additions like CNR0-4 from *eddy* and DWI Contrast from DSI Studio. The DWI time-series were resampled to AC-PC orientation, generating a *preprocessed DWI run in AC-PC space* with 1.7mm isotropic voxels.

## Supplementary Files: TOPUP and eddy settings

{

"flm": "quadratic",

"slm": "none",

"fep": false,

"interp": "spline",

"nvoxhp": 1000,

"fudge_factor": 10,

"dont_sep_offs_move": false,

"dont_peas": false,

"niter": 5,

"method": "jac",

"repol": true,

"num_threads": 1,

"is_shelled": true,

"use_cuda": false,

"cnr_maps": true,

"residuals": false,

"output_type": "NIFTI_GZ",

"estimate_move_by_susceptibility": true,

"args": ""

}
