# Supplementary material for: Diffusion MRI processing in the HEALthy Brain and Child Development study: Innovations and applications
Source: Dev Cogn Neurosci. 2026 Oct 15;82:101838. doi: 10.1016/j.dcn.2026.101838 (PMC13734816; doi:10.1016/j.dcn.2026.101838)
Supplement: Supplementary file 10 [file mmc2.docx]

Scanner Distribution Across Sites

| **Site** | **Manufacturer** | **Software Version** | **N Sessions** |
| --- | --- | --- | --- |
| hbcdsite77 | GE | 29\LX\DV29.1_R02_2131.a | 25 |
| hbcdsite35 | GE | 29\LX\DV29.1_R02_2131.a | 13 |
| hbcdsite35 | GE | 29\LX\DV29.1_R04_2313.a | 4 |
| hbcdsite26 | Philips | 5.9.0\5.9.0.0 | 38 |
| hbcdsite04 | Philips | 5.9.0\5.9.0.0 | 24 |
| hbcdsite52 | Philips | 5.7.1\5.7.1.0 | 21 |
| hbcdsite07 | Philips | 5.7.1\5.7.1.2 | 15 |
| hbcdsite57 | Philips | 5.7.1\5.7.1.3 | 3 |
| hbcdsite72 | Siemens | syngo MR XA30 | 47 |
| hbcdsite43 | Siemens | syngo MR XA30 | 43 |
| hbcdsite69 | Siemens | syngo MR XA30 | 40 |
| hbcdsite20 | Siemens | syngo MR XA30 | 38 |
| hbcdsite70 | Siemens | syngo MR XA30 | 34 |
| hbcdsite48 | Siemens | syngo MR XA30 | 29 |
| hbcdsite84 | Siemens | syngo MR XA30 | 26 |
| hbcdsite27 | Siemens | syngo MR XA30 | 22 |
| hbcdsite78 | Siemens | syngo MR XA30 | 21 |
| hbcdsite93 | Siemens | syngo MR XA30 | 21 |
| hbcdsite53 | Siemens | syngo MR XA30 | 16 |
| hbcdsite09 | Siemens | syngo MR XA30 | 11 |
| hbcdsite64 | Siemens | syngo MR XA30 | 11 |
| hbcdsite29 | Siemens | syngo MR XA30 | 7 |
| hbcdsite02 | Siemens | syngo MR XA30 | 4 |
| hbcdsite06 | Siemens | syngo MR XA30 | 4 |
| hbcdsite28 | Siemens | syngo MR XA30 | 4 |
| hbcdsite42 | Siemens | syngo MR XA30 | 4 |
| hbcdsite79 | Siemens | syngo MR XA30 | 4 |
