# Supplementary material for: Diffusion MRI processing in the HEALthy Brain and Child Development study: Innovations and applications
Source: Dev Cogn Neurosci. 2026 Oct 15;82:101838. doi: 10.1016/j.dcn.2026.101838 (PMC13734816; doi:10.1016/j.dcn.2026.101838)
Supplement: Supplementary file 11 [file mmc3.docx]

| Model | Parameter | Description | Shells | Category |
| --- | --- | --- | --- | --- |
| **qsirecon-DIPYDKI** | | | | |
| dki | ad | Axial diffusivity | Full | Complex Diffusivity |
| dki | ak | Axial kurtosis | Full | Diffusion Kurtosis |
| dki | fa | Fractional anisotropy | Full | Diffusion Anisotropy |
| dki | kfa | Kurtosis fractional anisotropy | Full | Diffusion Kurtosis |
| dki | md | Mean diffusivity | Full | Complex Diffusivity |
| dki | mk | Mean kurtosis | Full | Diffusion Kurtosis |
| dki | mkt | Mean kurtosis tensor | Full | Diffusion Kurtosis |
| dki | rd | Radial diffusivity | Full | Complex Diffusivity |
| dki | rk | Radial kurtosis | Full | Diffusion Kurtosis |
| **qsirecon-DSIStudio** | | | | |
| gqi | gfa | Generalized fractional anisotropy | Full | Diffusion Anisotropy |
| gqi | iso | Isotropic diffusion | Full |  |
| gqi | qa | Quantitative anisotropy | Full | Diffusion Anisotropy |
| tensor | ad | Axial diffusivity (first eigenvalue) from a tensor fit | Inner | Mean Diffusivity |
| tensor | fa | Fractional anisotropy from a tensor fit | Inner | Diffusion Anisotropy |
| tensor | ha | Helix angle from tensor fit | Inner |  |
| tensor | md | Mean diffusivity from a tensor fit | Inner | Mean Diffusivity |
| tensor | rd | Radial diffusivity from a tensor fit | Inner | Mean Diffusivity |
| tensor | rd1 | Lambda 2 (second eigenvalue) from a tensor fit | Inner | Mean Diffusivity |
| tensor | rd2 | Lambda 3 (third eigenvalue) from a tensor fit | Inner | Mean Diffusivity |
| tensor | txx | Tensor fit txx | Inner |  |
| tensor | txy | Tensor fit txy | Inner |  |
| tensor | txz | Tensor fit txz | Inner |  |
| tensor | tyy | Tensor fit tyy | Inner |  |
| tensor | tyz | Tensor fit tyz | Inner |  |
| tensor | tzz | Tensor fit tzz | Inner |  |
| **qsirecon-TORTOISE_model-MAPMRI** | | | | |
| mapmri | ng | Non-Gaussianity | Full | Diffusion Kurtosis |
| mapmri | ngpar | Non-Gaussianity parallel | Full | Diffusion Kurtosis |
| mapmri | ngperp | Non-Gaussianity perpendicular | Full | Diffusion Kurtosis |
| mapmri | pa | Propagator anisotropy | Full | Diffusion Kurtosis |
| mapmri | path | Thresholded propagator anisotropy | Full | Diffusion Kurtosis |
| mapmri | rtap | Return to axis probability | Full | Mean Diffusivity |
| mapmri | rtop | Return to origin probability | Full | Mean Diffusivity |
| mapmri | rtpp | Return to plane probability | Full | Mean Diffusivity |
| tensor | ad | Axial diffusivity | Inner | Mean Diffusivity |
| tensor | am | A0 from a tensor fit | Inner |  |
| tensor | fa | Fractional anisotropy from a tensor fit | Inner | Diffusion Anisotropy |
| tensor | li | Lattice index | Inner | Diffusion Anisotropy |
| tensor | rd | Radial diffusivity from a tensor fit | Inner | Mean Diffusivity |
| **qsirecon-TORTOISE_model-tensor** | | | | |
| tensor | ad | Axial diffusivity | Full | Mean Diffusivity |
| tensor | am | A0 from a tensor fit | Full |  |
| tensor | fa | Fractional anisotropy from a tensor fit | Full | Diffusion Anisotropy |
| tensor | li | Lattice index | Full | Diffusion Anisotropy |
| tensor | rd | Radial diffusivity from a tensor fit | Full | Mean Diffusivity |
