# Supplementary material for: Diffusion MRI processing in the HEALthy Brain and Child Development study: Innovations and applications
Source: Dev Cogn Neurosci. 2026 Oct 15;82:101838. doi: 10.1016/j.dcn.2026.101838 (PMC13734816; doi:10.1016/j.dcn.2026.101838)
Supplement: Supplementary file 12 [file mmc4.docx]

CNR Statistics by Scanner Model and b-value

| **Manufacturer** | **Manufacturer Model Name** | **b-value** | **Mean CNR** | **Median CNR** | **Std CNR** |
| --- | --- | --- | --- | --- | --- |
| GE | DISCOVERY MR750 | b=3000 | 0.556 | 0.555 | 0.142 |
| Philips | Achieva dStream | b=3000 | 1.036 | 1.044 | 0.203 |
| Philips | Ingenia Elition X | b=3000 | 0.979 | 1.005 | 0.183 |
| Philips | MR 7700 | b=3000 | 0.742 | 0.747 | 0.180 |
| Siemens | MAGNETOM Prisma | b=3000 | 1.637 | 1.618 | 0.631 |
| Siemens | MAGNETOM Prisma Fit | b=3000 | 1.450 | 1.431 | 0.414 |
| GE | DISCOVERY MR750 | b=2000 | 0.544 | 0.548 | 0.165 |
| Philips | Achieva dStream | b=2000 | 1.071 | 1.147 | 0.310 |
| Philips | Ingenia Elition X | b=2000 | 1.147 | 1.209 | 0.275 |
| Philips | MR 7700 | b=2000 | 0.764 | 0.837 | 0.219 |
| Siemens | MAGNETOM Prisma | b=2000 | 1.934 | 2.050 | 0.582 |
| Siemens | MAGNETOM Prisma Fit | b=2000 | 1.683 | 1.735 | 0.553 |
| GE | DISCOVERY MR750 | b=1000 | 0.320 | 0.340 | 0.103 |
| Philips | Achieva dStream | b=1000 | 0.662 | 0.562 | 0.345 |
| Philips | Ingenia Elition X | b=1000 | 0.892 | 0.850 | 0.369 |
| Philips | MR 7700 | b=1000 | 0.605 | 0.626 | 0.255 |
| Siemens | MAGNETOM Prisma | b=1000 | 1.316 | 1.387 | 0.570 |
| Siemens | MAGNETOM Prisma Fit | b=1000 | 1.146 | 1.161 | 0.573 |
| GE | DISCOVERY MR750 | b=500 | 0.176 | 0.171 | 0.063 |
| Philips | Achieva dStream | b=500 | 0.318 | 0.285 | 0.155 |
| Philips | Ingenia Elition X | b=500 | 0.375 | 0.372 | 0.145 |
| Philips | MR 7700 | b=500 | 0.285 | 0.311 | 0.102 |
| Siemens | MAGNETOM Prisma | b=500 | 0.564 | 0.599 | 0.253 |
| Siemens | MAGNETOM Prisma Fit | b=500 | 0.473 | 0.466 | 0.249 |
